# Knowledge brokering: Exploring the process of transferring knowledge into action

**DOI:** 10.1186/1472-6963-9-12

**Published:** 2009-01-16

**Authors:** Vicky L Ward, Allan O House, Susan Hamer

**Affiliations:** 1 Leeds Institute of Health Sciences, University of Leeds, Leeds, UK; 2 Faculty of Medicine and Health, University of Leeds, Leeds, UK

## Abstract

**Background:**

There are many theories about knowledge transfer but there are few clear descriptions of knowledge transfer interventions or the processes they involve. This failure to characterise structure and process in proposed KT interventions is a major barrier to the design and implementation of evaluations of particular KT strategies. This study is designed to provide a detailed description of the processes involved in a knowledge transfer intervention and to develop and refine a useful model of the knowledge transfer process.

**Methods and design:**

This research is taking a sociological approach to investigating the process of knowledge transfer. The approach is designed to articulate the broad components of the knowledge transfer process and to test these against evidence from case study sites. The research falls into three phases. First, we have carried out a literature review to produce a theoretical framework of the knowledge transfer process. This involved summarising, thematically analysing and synthesising evidence from the literature. Second, we are carrying out fieldwork in a mental health setting based on the application of a knowledge brokering intervention. The intervention involves helping participants identify, refine and reframe their key issues, finding, synthesising and feeding back research and other evidence, facilitating interactions between participants and relevant experts and transferring information searching skills to participants. Finally, we are using the observations of the knowledge broker and interviews with participants to produce narratives of the brokering process. The narratives will be compared in order to identify evidence which will confirm, refute or revise each of the broad components of the knowledge transfer process. This comparison will enable us to generate a refined framework of knowledge transfer which could be used as a basis for planning and evaluating knowledge transfer interventions.

**Discussion:**

This study will provide an opportunity for a detailed description of a knowledge transfer intervention and the processes which are involved. Our approach is also designed to enable us to develop and refine a useful model of the knowledge transfer process. We believe that it will significantly enhance the growing body of knowledge about knowledge transfer.

## Background

This study is designed to provide information about the process of transferring knowledge into action. It focuses on knowledge brokering, one of the proposed methods of knowledge transfer. Our aim is to develop and refine a useful model of the knowledge transfer process through documenting the processes involved in knowledge brokering.

### The importance of knowledge transfer

Doing health research is costly and time-consuming but often the results are acted upon slowly or not at all. The gap between what is known and what is done leads not only to the under-use of effective treatments but also to the incorrect use of treatments and the over-use of unhelpful or unproven treatments. This results in poor health outcomes, health inequities and wasted time and money [1,2].

The realisation that failing to use research findings in healthcare has a negative impact on patient care has led to an increased emphasis on finding and using appropriate ways of transferring research into practice. This process is commonly referred to as 'knowledge transfer' but a number of other terms have been used including knowledge translation, knowledge exchange, research utilization, innovation spread and linkage and exchange [3]. In recent years knowledge transfer has become a significant focus for policy makers and researchers and activities which link research-generated evidence to policy and practice are now starting to be mandated by research councils [4,5].

Knowledge transfer is recognised as a complex and messy process which goes beyond the one-way push of information from researchers to decision makers [3]. Methods include encouraging researchers and decision makers to work in partnership on planning, disseminating and implementing research, ensuring that research is relevant, timely and can be readily applied to the users' context, and identifying and supporting opinion leaders and champions within the academic and practice environments.

### Knowledge brokering

One of the main obstacles to transferring research into practice is believed to be the presence of a gap between those who produce research and those who use it. Decision makers and researchers inhabit different worlds, each of which is based on varying beliefs, values and practices [6]. Given these complexities and difficulties, it is sometimes thought that neither researchers nor decision makers are best placed to drive the translation, transfer and implementation of health research evidence. A proposed solution is to use 'knowledge brokers' whose job is to facilitate the transfer of research and other evidence between researchers and practitioners. Positioned at the interface between the worlds of researchers and decision makers, they are seen as the human force behind knowledge transfer whose tasks include finding, assessing and interpreting evidence, facilitating interaction and identifying emerging research questions [7].

Three different approaches to brokering have been identified within the literature. The first relates to the creation, diffusion and use of knowledge and brokering is seen as a way of facilitating or managing these activities. In this approach brokers act as 'knowledge managers'. In the second, brokering focuses on the interface between the "creators" and "users" of knowledge and seeks to foster links between the two. In this approach brokers act as linkage agents. Finally, in the third approach brokering is designed to enhance access to knowledge by providing training to knowledge users which may lead to positive social outcomes. In this context brokers act as capacity builders [8]. These functions of brokering have become widely accepted and form the basis for much of the practical work on knowledge brokering in the public sector.

### Issues associated with knowledge transfer

Whilst there is widespread agreement about the importance of transferring knowledge into action we are still far from knowing what works, in which setting and with whom [9]. This is due to several reasons.

First, studies in areas such as behaviour change and research utilisation have suggested that no single approach is effective in all circumstances. Instead, strategies such as the distribution of educational materials, courses, opinion leaders and feedback all show mixed effects on the rate at which evidence is translated into action [10].

Second, previous studies have given little attention to broad explanations of the process of transferring knowledge into action. Instead they have tended to assume that a relatively narrow range of factors affect the implementation of research evidence. These include characteristics of the research such as rigour and credibility, characteristics of the organisation such as size and innovativeness and characteristics of the intervention such as timing and intensity [11-13]. Such deterministic approaches presume that both the knowledge itself and the contexts in which it is implemented are uniform and tend not to acknowledge the complexity of the process.

Third, descriptions of the processes involved in different knowledge transfer interventions, such as knowledge brokering, are vague. The systematic use of knowledge transfer methods is uncommon and a recent review identified only eighteen studies which described the implementation of a specific knowledge transfer mechanism [14]. This means that we do not know much about the processes involved in different knowledge transfer interventions.

Finally, there is a lack of suitable tools for planning and evaluating knowledge transfer activities. Whilst a clearly articulated model or framework can form the basis for planning, delivering and evaluating knowledge transfer interventions, the number of alternative theories has become unwieldy. Recent reviews have identified as many as 63 different theories or models of knowledge transfer across fields as diverse as healthcare, social care and management [14,15]. This means that it is difficult for researchers and managers to choose which model to use as an aid to planning and evaluating knowledge transfer interventions. In addition, many of the models remain largely unrefined and untested meaning that their suitability as tools for designing and evaluating interventions is unknown.

### Purpose and objectives of the study

The purpose of this research is to gain a better understanding of the processes involved in transferring knowledge into action. Our objectives are to clearly describe the processes involved in a knowledge brokering intervention and to produce a framework of the knowledge transfer process which can be used for planning and evaluating knowledge transfer activities.

### Conceptual framework

Our conceptual framework for this research is based on aspects of the sociological models of diffusion and innovation. From these models we have conceptualised knowledge transfer as a complex social activity which involves the activities of many communities, is influenced and moulded by the belief systems and analytical or creative instincts of potential users and encompasses the reinvention, proliferation and reimplementation of ideas, the fluid engagement of multiple entrepreneurs and an expanding and contracting network of stakeholders who converge and diverge [16,17].

## Methods and design

### Research design

This research is taking a realist approach to investigating the process of knowledge transfer [18]. The realist approach is designed to articulate and test theories across different contexts with the end product of the inquiry being a better understanding of which ideas work for whom, in which contexts, and why. Its value as a way of examining complex, unstable, nonlinear processes has been recognised, particularly within healthcare [19,20]. In this study we are using the approach to articulate the broad components of the knowledge transfer process and to test these against evidence from case study sites.

Our research has three distinct phases. First we used a review of the literature to produce a theoretical framework of the knowledge transfer process. This phase of the research is complete and the methods used are therefore described retrospectively below. Second, we are carrying out fieldwork in three sites based on the application of knowledge brokering. We are using a participant observation methodology where the knowledge broker is also responsible for researching her own processes. The fieldwork will enable us to document the processes involved in knowledge brokering and gather evidence for the theoretical components of the knowledge transfer process. This phase of the research is ongoing. Finally, we will use the fieldwork data to confirm, refute and revise the components of knowledge transfer and produce a revised framework of the knowledge transfer process. Our output will be a framework of the knowledge transfer process which can be used to produce guidance for researchers and decision makers on planning knowledge transfer interventions.

### Phase 1 – Theoretical framework

The theoretical framework was designed to articulate the broad components which seem crucial to the process of transferring knowledge into action. To produce the framework, we carried out a review of the literature using a narrative approach [21]. This involved summarizing, thematically analyzing and synthesizing evidence from two types of papers; those which reviewed or summarised the knowledge transfer literature and those which developed, evaluated or utilized knowledge transfer theories or models. Articles were identified by searching a range of databases and through a process of snowballing (i.e. references of references). We continued to search for new articles until March 2008. Through the detailed reading of 193 papers and reports we identified 28 different models which explained all or part of the knowledge transfer process. We subjected the models to a thematic analysis to identify a) the individual components of the knowledge transfer process and b) the type of processes used when transferring knowledge into action. The results of our thematic analysis and further details about the development of the theoretical framework can be found elsewhere [22].

### Phase 2 – Fieldwork

The second phase of the research involves implementing a knowledge brokering intervention as a way of documenting the processes involved in knowledge brokering and gathering evidence for broad components of the knowledge transfer process. This phase of the research is currently ongoing.

#### Participants

We are carrying out three case studies with participants from a mental health trust. Participants are all members of teams who want to be able to use research and other evidence in the planning, delivery or evaluation of their services.

Participants were recruited with the help of senior managers within the Trust who circulated a briefing to colleagues about the aims of the research. The briefing invited contact from individuals and teams who would welcome the opportunity to be involved in an intervention designed to link research with practice. We used an opt-in process whereby we did not contact teams directly but waited for them to contact us.

We were contacted by three teams and held an initial meeting with each to discuss the research project and the ways in which they wanted to use research and other evidence in their practice. This helped us to identify a series of initial questions or issues which participants wanted to address. One team wanted to find the best ways of implementing routine outcome measurement across their service, one team wanted to design and run a new service for their clients and one team wanted to provide information and advice to colleagues about how to offer a range of psychological therapies. As our intervention was designed to be tailored to the needs of each team, we produced and agreed an individual research protocol for each case study. Before proceeding with the intervention we obtained verbal consent from each service management/delivery team.

#### Intervention

Our intervention involves the use of 1 individual who is acting as knowledge broker with each of the teams. The precise activities of the broker are dependent upon the context and individual needs of the participants, as the literature is clear that assessing context is vital to choosing appropriate ways of transferring research and other evidence into practice [2,7]. However, we expect that the brokering tasks will include: helping participants identify, refine and reframe their key issues, questions and needs; finding, synthesising and feeding back relevant research and other evidence; finding appropriate experts to inform and assist the participants; facilitating interactions and mediating between participants and relevant experts; and transferring information searching and other skills to participants. We envisage that this will not be a one-off process, but will continue through multiple cycles as additional questions are identified and refined. The tasks will be carried out using a number of mechanisms including formal and informal meetings with participants, discussion forums, priority setting exercises and the targeted dissemination of information.

#### Data collection

Data collection will focus on documenting the processes involved in knowledge brokering. Two stages of data collection will be undertaken: direct participant observation and narrative interviews.

During the participant observation stage, the broker will keep a reflective field journal for each case study. This will include a diary of tasks and activities, comprehensive records of all electronic, telephone and face-to-face discussions and reflections on the progress and processes involved in the case study. Drawing on the work of Vaughan, the field journals will be used to produce chronological reconstructions of the brokering process [23]. These will then be used to inform the narrative interviews.

Narrative interviews will be carried out with participants from each case study site. This approach will involve inviting interviewees to tell stories about the process and content of knowledge brokering from their own perspective [24]. Interviews will focus on the activity of brokering rather than individuals' opinions of the outcomes of brokering. An interview guide will be produced which will contain a series of prompts. These will be based on the chronological reconstruction of brokering at the site and will focus on some of the episodes which seem particularly important to the process of brokering and those which do not seem to be explained by our theoretical framework.

### Phase 3 – Data analysis & synthesis

The third phase of the research involves using the fieldwork data to confirm, refute and revise our theoretical framework of knowledge transfer.

We will begin by using field journals and interview transcripts to generate rich narratives of the brokering process at each site. Each narrative will be based on the chronological reconstruction of brokering but will also contain further contextual details about the process and content of knowledge brokering at the site.

We will then carry out a comparative analysis [25] of brokering narratives using the theoretical framework developed during the first phase of the research. Focusing on the similarities and differences in brokering narratives will enable us to identify evidence which will either confirm or refute each of the five components of the knowledge transfer framework and to identify evidence which lies outside these components.

The results of the comparative analysis will be used to generate a refined, more nuanced framework of knowledge transfer which will contain further details about how the process occurs in different contexts and could be used as a basis for planning and evaluating knowledge transfer interventions.

### Research procedure and timeline

The study will proceed in four stages.

1. Theoretical framework (August 2007–March 2008)

• Perform literature search

• Thematically analyse literature

• Synthesise into theoretical framework

2. Fieldwork (January 2008–July 2009)

• Recruit participants

• Agree protocol for each site

• Participant observation of brokering activities

• Produce chronological reconstructions of brokering

• Narrative interviews with all participants

3. Data analysis & synthesis (January 2009–October 2009)

• Transcription of the interviews

• Produce brokering narratives

• Comparative analysis of the narratives

• Produce revised framework of knowledge transfer

4. Dissemination (December 2008–January 2010)

• Produce research publications and presentations

• Organise expert workshop on knowledge transfer and knowledge brokering

• Produce a project website

• Produce a full report, lay report and executive summary

• Discuss and agree suitable dissemination routes with mental health trust managers

### Ethical approval

The study has received ethical approval from South Humber NHS Research Ethics Committee.

## Discussion

This study is taking an innovative approach to investigating knowledge transfer as a complex social activity. Evaluating complex interventions has been the focus of much discussion but the focus has usually been on finding ways of standardising and measuring individual elements [26]. Realism provides an alternative approach which focuses on how and why interventions work in different contexts. This research will provide the opportunity to see how realism can be applied to the evaluation of complex interventions.

Many previous studies have aimed to explain and determine the outcomes of knowledge transfer interventions by focusing on a narrow range of causal factors. This focus has not provided a broad enough bases for describing the processes involved in knowledge transfer interventions. This study will provide an opportunity for a detailed description of a knowledge transfer intervention and the processes which are involved.

Although there are a large number of alternative models and theories of knowledge transfer, the majority have not been refined or tested and as such cannot form the basis for designing and evaluating knowledge transfer interventions. In contrast, our approach is designed to enable us to develop and refine a useful model of the knowledge transfer process and will significantly enhance the growing body of knowledge about knowledge transfer.

## Competing interests

The authors declare that they have no competing interests.

## Authors' contributions

All authors participated in the conception and design of the study. VW carried out the literature review and thematic analysis and drafted the manuscript. All authors read and approved the final manuscript.

## Pre-publication history

The pre-publication history for this paper can be accessed here:



## References

[B1] World Health Organization (2004). World Report on Knowledge for Better Health: Strengthening health systems.

[B2] Berwick DM (2003). Disseminating Innovations in Health Care. Journal of the American Medical Association.

[B3] Graham ID, Logan J, Harrison MB, Straus SE, Tetroe J, Caswell W (2006). Lost in knowledge translation: Time for a map?. Journal of Continuing Education in the Health Professions.

[B4] Darzi Lord (2007). Our NHS, Our Future: NHS next stage review interim report.

[B5] Tetroe JM, Graham ID, Foy R, Robinson N, Eccles MP, Wensing M (2008). Health Research Funding Agencies' Support and Promotion of Knowledge Translation: An International Study. Milbank Quarterly.

[B6] Caplan N (1979). The Two-Communities Theory and Knowledge Utilization. American Behavioral Scientist.

[B7] CHSRF (2003). The theory and practice of knowledge brokering in Canada's health system.

[B8] Oldham G, McLean R (2007). Approaches to Knowledge-Brokering. http://www.iisd.org/pdf/2001/networks_knowledge_brokering.pdf.

[B9] Lomas J (1997). Improving Research Dissemination and Uptake in the Health Sector: Beyond the sound of one hand clapping.

[B10] Haines A, Kuruvilla S, Borchert M (2004). Bridging the implementation gap between knowledge and action for health. Bulletin of the World Health Organization.

[B11] Grimshaw JM, Thomas RE, MacLennan G, Fraser C, Ramsay CR, Vale L (2004). Effectiveness and efficiency of guideline dissemination and implementation strategies. Health Technology Assessment.

[B12] Grol R (2001). Successes and Failures in the Implementation of Evidence-Based Guidelines for Clinical Practice. Med Care.

[B13] Cabana MD, Rand CS, Powe NR, Wu AW, Wilson MH, Abboud P-AC (1999). Why Don't Physicians Follow Clinical Practice Guidelines?: A Framework for Improvement. Journal of the American Medical Association.

[B14] Mitton C, Adair CE, McKenzie E, Patten SB, Perry BW (2007). Knowledge Transfer and Exchange: Review and Synthesis of the Literature. The Milbank Quarterly.

[B15] Graham ID, Tetroe J (2007). Some theoretical underpinnings of knowledge translation. Academic Emergency Medicine.

[B16] Ven AH Van de, Polley DE, Garud R, Venkataraman S (1999). The Innovation Journey.

[B17] Rogers EM (2003). Diffusion of Innovations.

[B18] Pawson R (2006). Evidence-based Policy: A realist perspective.

[B19] Berwick DM (2008). The Science of Improvement. Journal of the American Medical Association.

[B20] Dickinson H (2006). The evaluation of health and social care partnerships: an analysis of approaches and synthesis for the future. Health and Social Care in the Community.

[B21] Mays N, Pope C, Popay J (2005). Systematically reviewing qualitative and quantitative evidence to inform management policy-making in the health field. Journal of Health Services Research & Policy.

[B22] Ward V, House A, Hamer S Developing a framework for transferring knowledge into action: a thematic analysis of the literature. Journal of Health Services Research & Policy.

[B23] Vaughan D (1996). The Challenger Launch Decision: Risky technology, culture and deviance at NASA.

[B24] Wengraf T (2001). Qualitative Research Interviewing.

[B25] Ragin CC (1987). The Comparative Method.

[B26] MRC (2000). A framework for development and evaluation of RCTs for complex interventions to improve health. Medical Research Council.

